# Evidence of prevailing practice of home slaughter in Iran revealed by bioeconomic modeling of small ruminants slaughtered in and outside registered abattoirs

**DOI:** 10.1371/journal.pone.0337839

**Published:** 2026-02-02

**Authors:** Mohammad Ebrahimipour, Mehdi Borhani, Omid Dayani, Majid Fasihi Harandi

**Affiliations:** 1 Research Center for Hydatid Disease in Iran, Institute of Infectious Diseases and Tropical Medicine, Afzalipour School of Medicine, Kerman University of Medical Sciences, Kerman, Iran; 2 State Key Laboratory of Pathogenesis, Prevention and Treatment of High Incidence Diseases in Central Asia, Xinjiang Medical University, Urumqi, China; 3 Department of Animal Science, Faculty of Agriculture, Shahid Bahonar University of Kerman, Kerman, Iran; Makerere University College of Natural Sciences, UGANDA

## Abstract

Home slaughter seems to be a prevailing practice in developing countries, and presents a potential public health risk and animal welfare problem for the societies all over the world. Nevertheless, the nature and extent of this practice is poorly understood in many countries. The objective of this study was to estimate the number of sheep and goats slaughtered outside registered abattoirs in Iran and to discuss the possible determinants of this practice. Number of live and slaughtered animals, human population, and per capita red meat consumption were extracted from FAOSTAT and the Statistical Center of Iran (SCI). Per capita red meat consumption and bio-economic modeling of flock compositions were used to estimate non-abattoir slaughter numbers. Based on per capita meat consumption and the bio-economic models, it was estimated that 7,937,725 (42.3%) and 12,809,170 (54.1%) of sheep and goats were slaughtered either at home or in unregulated abattoirs during 2017. Home slaughter is a neglected problem in numerous countries and communities. Additional studies are needed to clarify the nature and extent of this human and livestock health challenge. An integrated One Health surveillance system is needed to address this practice in developing countries.

## Introduction

Population growth, urbanization, economic progress, and changing consumer preferences contribute to an increasing demand for meat and other livestock products globally [1,2]. Livestock have economic, social, and cultural roles in rural households and contribute to local community income and wellbeing [3]. Animal husbandry also contributes to food security, employment, soil productivity, sustainable agricultural production, and religious traditions. Approximately 1.3 billion people worldwide work in some aspect of livestock production and three-quarters of poor rural households worldwide possess animals as a main source of their livelihood [2].

Slaughtering livestock for personal consumption has been practiced since ancient times. As population sizes grew, centralized abattoirs became popular since they increased efficiency [4]. However, home/on-farm slaughtering remains, particularly in lower income countries. Many centralized abattoirs in more remote areas of low income and lower middle income countries are also considered sub-standard [5]. The slaughter of livestock in poorly equipped abattoirs is an important issue that directly threatens human health globally [6,7]. In many developing countries, centralized abattoirs themselves are considered sub-standard, often lacking modern infrastructure, adequate chilling systems, or proper waste disposal. While these deficiencies compromise hygienic standards and animal welfare, it is important to note that abattoirs nonetheless operate under veterinary supervision. Meat produced in registered centralized abattoirs undergoes veterinary inspection under the authority of the Iran Veterinary Organization (IVO). Veterinarians and trained meat hygiene inspectors are responsible for ante-mortem and post-mortem inspection, removal of diseased carcasses or viscera, and certification of meat for human consumption. The presence of veterinarians and meat inspectors ensures that diseased livestock carcasses can be detected and removed from the food chain, a safeguard entirely absent in home slaughter that takes place entirely outside this regulatory framework. Therefore, even sub-standard abattoirs remain far preferable to unsupervised home slaughter from a public health perspective [8].

Close contact with animals at abattoirs and other slaughtering sites presents a potential risk of zoonotic infections, most notably brucellosis and foodborne parasitic diseases such as cystic echinococcosis (CE). Although less common, other zoonoses such as Crimean–Congo hemorrhagic fever (CCHF) and anthrax have also been reported in association with livestock handling or consumption of contaminated meat in Iran [9–13]. Numerous other bacterial, viral, and parasitic foodborne pathogens have been identified [14–16]. According to the World Health Organization (WHO) and the United States Centers for Diseases Control and Prevention (CDC), foodborne pathogens cause an estimated 76 million illnesses, 325,000 hospitalizations, and 5000 deaths globally per year. The cornerstone of control and prevention of foodborne illnesses is meat inspection [9,17]. In Iran, the presence of different livestock species, traditional animal husbandry practices, and traditional food preparation and consumption practices has resulted in a number of common foodborne diseases such as *Brucella*, and *Salmonella* infections [18–20]. Such zoonotic risks highlight the direct consequences of slaughtering animals outside regulated facilities.

In addition to zoonotic infections, food safety concerns are central to the issue of home slaughter. The absence of veterinary inspection removes a critical safeguard against contaminated or unsafe meat entering the food chain [21]. In Iran, this risk is particularly relevant for *Brucella* spp., which remain endemic in livestock as well as other pathogens, including *Salmonella*, that may also contaminate meat when slaughter and butchering are performed under unhygienic household conditions. It is important to emphasize that food safety hazards, alongside zoonotic disease transmission, are a major consequence of unregulated slaughter in Iran [18].

Beyond public health and food safety risks, animal welfare is an additional concern in home slaughter. In most household or roadside settings, animals are slaughtered without pre-slaughter stunning, which leads to prolonged stress, pain, and distress. In addition, improper restraint, handling in front of other animals, and lack of trained personnel further compromise welfare and may also affect meat quality. The World Organization for Animal Health (WOAH), in its Terrestrial Animal Health Code, emphasizes that all animals should be handled calmly, restrained humanely, and rendered insensible before bleeding [22]. However, pre-slaughter stunning particularly in certain religious contexts is not permitted and is rarely observed in slaughter practice in Iran. Therefore, home slaughter poses ethical, professional, and public health challenges simultaneously. Home slaughter is not only a public health and food safety issue but also an ethical and animal welfare challenge that requires attention alongside disease control and hygiene measures.

Livestock slaughtered outside of official abattoirs presents a possible health risk for abattoir workers and the consumers of their products [15]. Understanding livestock slaughtering practices outside of official abattoirs is essential for the management and control of zoonoses in endemic countries. Bio-economic models have been used to calculate the economic values of breeding sheep and goats across the globe [22–26]. The objectives of this study were to estimate the number of small ruminants slaughtered outside of registered abattoirs in Iran using per capita meat consumption and bio-economic modeling, and discuss possible motivations for this practice.

## Methods

The present study was approved by the Research Ethics Review Committee of Kerman University of Medical Sciences. In this study, the term “home slaughter” and/or “unregulated slaughter” refers specifically to the slaughter of livestock by households without official inspection or by unregistered small abattoirs operating outside any formal facility or veterinary supervision. Two methods, (a) per capita meat consumption vs. official slaughter data and (b) bioeconomic flock composition models, were applied to available data from Iran. We employed two complementary approaches in order to cross-validate estimates. This triangulation reduces reliance on a single method and increases confidence in the results.

### Estimation of sheep and goats slaughtered outside of official abattoirs based on per capita meat consumption

The United Nations Food and Agriculture Organization, FAOSTAT (https://www.fao.org/faostat/en/#data) interface and the Statistical Center of Iran (SCI) website (https://www.amar.org.ir) were queried to obtain the number of live and slaughtered sheep and goats, human population, and per capita red meat consumption. The average carcass weight for sheep and goats was 18.7 kg and 14.7 kg, respectively. Total combined slaughter weight for all sheep and goats officially slaughtered during 2017 was calculated by multiplying the mean slaughter weight of each species by the total numbers of officially slaughtered sheep and goats. The year 2017 was selected from FAOSTAT and SCI databases, as it provided the most recent and internally consistent datasets across both sources. Table 1 provides an overview of the data for sheep and goats extracted from FAOSTAT and SCI. Meat exports were excluded from HSs and HSg calculations, as official data show that exported sheep and goat meat from Iran is negligible and does not affect national consumption or slaughter estimates.

**Table 1:** Overview of primary data of sheep and goat extracted from FAOSTAT and statistical center of Iran, used for estimating home slaughter in Iran in 2017.

Variable	Livestock		Total
	Sheep	Goat	
**Live animal (No.)**	40,029,688	15,711,084	55,740,772
**Slaughtered animal, official (No.)**	8,609,118	2,223,110	10,832,228
**Average weight of carcass (kg)**	18.7	14.7	33.4
**Total slaughtered weight, official (kg)**	160,990,507	32,679,717	193,670,224
**Per capita meat consumption (per person per year, kg)**	3.412	0.77	4.182
**Total meat consumption (kg)**	272,708,433	61,543,228	334,251,661
**Slaughter at home and/or sub-standard abattoirs (kg)**	111,717,927	28,863,511	140,581,438
**Slaughter at home and/or sub-standard abattoirs (No.)**	5,974,221	1,963,504	7,937,725
**Total animals slaughtered at official and/or unregulated abattoirs (No.)**	14,583,339	4,186,614	18,769,953
**Proportion of home slaughter (%)**	41.0	40.5	42.3

The human papulation of Iran was 79,926,270 based on the latest population and housing censuses in 2016. In 2017, per capita red meat (cattle, sheep, goats, buffaloes, and camels) consumption was 10 kg. National abattoir data categorized by livestock species were used to calculate approximate per capita meat consumption for sheep and goats. Sheep and goat meat contributed 34.1% and 7.7% of the total meat production volume, respectively. Per capita meat consumption was estimated at 3.41 kg for sheep and 0.77 kg for goats during 2017. All data and the corresponding calculations were performed in an MS Excel spreadsheet.

To calculate the total estimated weight (kg) of sheep and goat meat produced outside of official slaughterhouses, the following formulas were applied:


$${𝐇𝐒}_{𝐒}=\left({𝐏𝐎𝐏}_{𝐡}\times {𝐏𝐂𝐂}_{𝐬}\right)-{𝐒𝐖}_{𝐚𝐬}$$

$${𝐇𝐒}_{𝐠}=\left({𝐏𝐎𝐏}_{𝐡}\times {𝐏𝐂𝐂}_{𝐠}\right)-{𝐒𝐖}_{𝐚𝐠}$$

where HS_s_ is the total home slaughter weight of sheep and HS_g_ is the total home slaughter weight of goats. POP_h_ is the human population of Iran, PCC_s_ and PCC_g_ are per capita meat consumption for sheep and goats, respectively, and SW_as_ and SW_ag_ are the official abattoir slaughter weights for sheep and goats, respectively. To estimate the number of sheep and goats slaughtered outside of official slaughterhouses, total home slaughter weights (HS_s_ and HS_g_) were divided by the average carcass weights of sheep and goats.

### Estimation of sheep and goats slaughtered outside of official abattoirs based on bio-economic modeling

Bio-economic modeling was also used to estimate home slaughter of sheep and goats in Iran. Values associated with sheep and goat flock composition obtained from several previous studies are shown in Table 2 [22–24,26]. Comparable productivity parameters for Iranian breeds are limited in the literature, therefore we used some of the studies conducted in non-Iranian contexts [27,28]. However, available reports suggest that reproductive and survival rates of Iranian small ruminants fall within similar ranges. Therefore, we adopted these parameters as representative estimates. Male and female sheep and goats of reproductive age were modeled for a one-year period and the numbers of animals and their offspring sold for slaughter were estimated after applying age- and sex-specific conception and survival rates. Offspring sex ratio was assumed to be 50% male and 50% female for both sheep and goats. To estimate total number of animals slaughtered at home and/or unofficial abattoirs, the official numbers of animals slaughtered in abattoirs was subtracted from the numbers of animals sold for slaughter, according to flock composition modeling.

**Table 2:** List of parameters and the values used for bio-economic modeling based on population balance and flock composition of sheep and goat [23,24,26–28].

Variable	Value	
	Sheep	Goat
**Reproducing frequency (per animal per year)**	1.5	1.5
**Twinning rate (%)**	9.0	18.0
**Conception rate (%)**	93	95
**Survival rate of reproducing females (%)**	90	90
**Survival rate at 3 months of age (%)**	80	82
**Survival rate at 12 months of age (%)**	85	88
**Surplus, male (%)**	97.85	98.06
**Surplus, female (%)**	57.45	34.07
**Culled reproducing male (%)**	50.00	53.00
**Culled reproducing female (%)**	12.00	26.00

A deterministic bioeconomic model was constructed to estimate offspring and demographic transitions in a reproductive livestock system. The base model was constructed using empirical parameters, such as the proportion of twinning females, reproduction rates, offspring per female per year, and survival rates at 3 and 12 months. The initial reproducing population was fixed at 379,738 males and 17,095,168 females for sheep and 149,042 males and 7,455,126 females for goats. Input values were informed by field data and expert knowledge. Intermediate variables such as the number of females with and without twinning, conception rates, and multipliers accounting for litter size and reproduction frequency were incorporated into the model as multiplicative factors in the calculation of offspring production.

**Fig 1:**
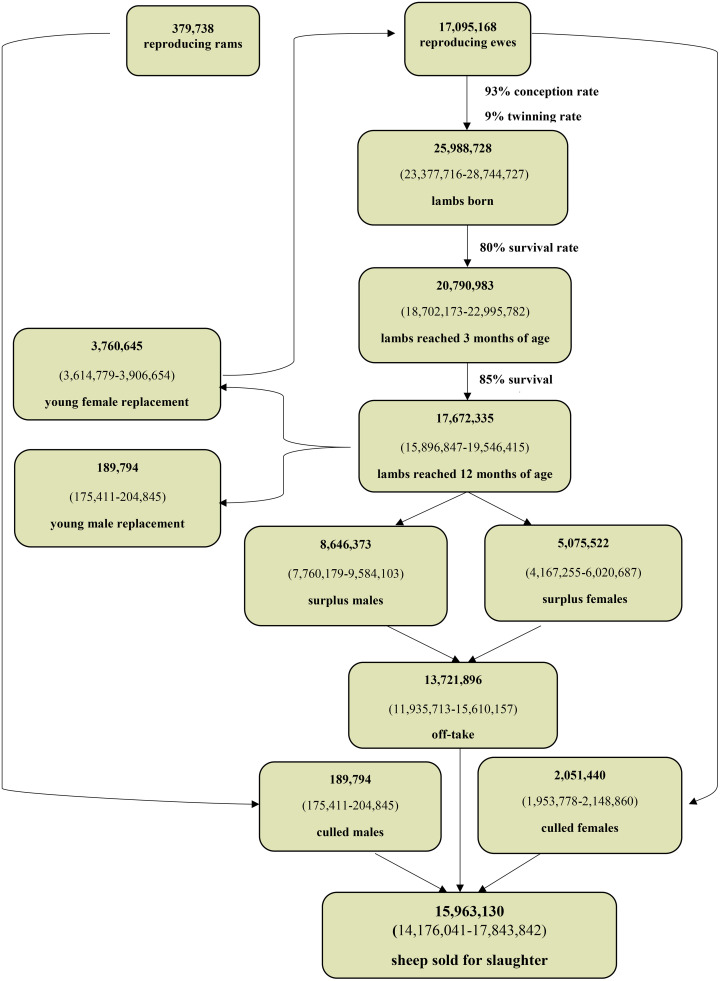
Flock composition dynamics and population balance model developed for sheep.

**Fig 2:**
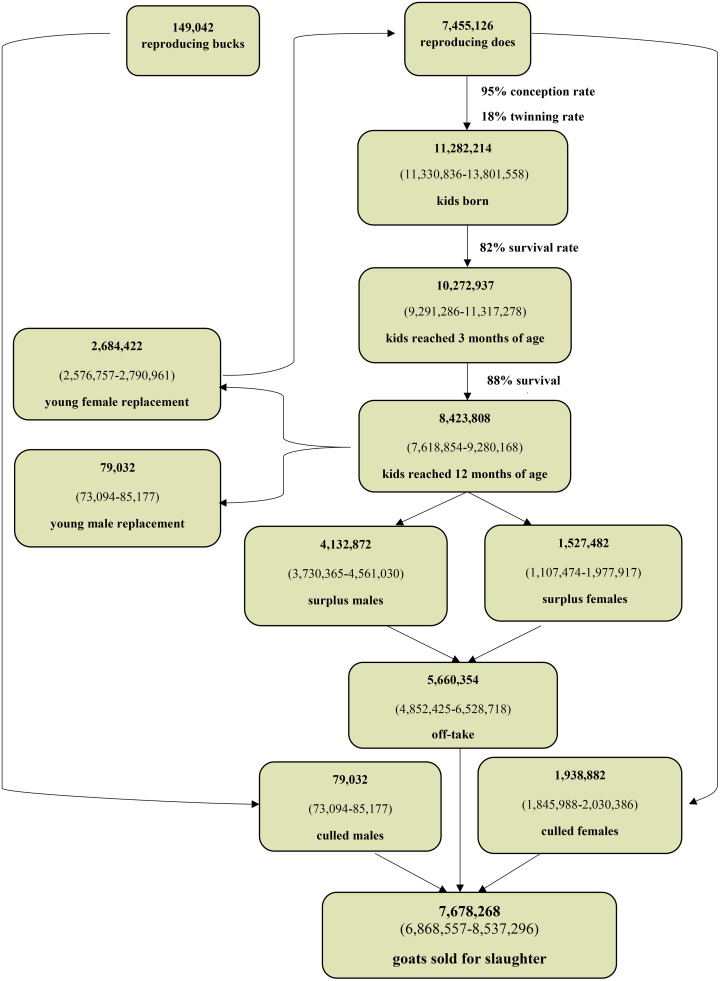
Flock composition dynamics and population balance model developed for goat.

To quantify uncertainty, we applied a Monte Carlo simulation with 10,000 iterations, drawing from uniform distributions with ±5% uncertainty around key parameters. Outputs included the total number of offspring born, survival to key ages, replacement needs, and surpluses. Confidence intervals (CIs) were derived using empirical quantiles (2.5th, 50th, and 97.5th percentiles). All simulations and analyses were conducted in R (version 4.3.1). Figs 1 and 2 show the flock composition dynamics and population balance models developed for sheep and goat, respectively.

## Results

Based on red meat consumption data, 7,937,725 sheep and goats were estimated to have been slaughtered either at home or in unregulated abattoirs during 2017. These 5,974,221 sheep and 1,963,504 goats are in addition to the reported 10,832,228 sheep and goats officially slaughtered in Iranian abattoirs that year, representing 42.3% of total slaughtered animals (Table 1). In comparison, using the bio-economic model, 7,354,012 sheep and 5,455,158 goats were estimated to have been unofficially slaughtered in 2017 for a total of 12,809,170 animals. This value suggests that 54.1% of slaughtered animals are processed at home or in unsanctioned abattoirs. Figs 1 and 2 show the number of animals sold for slaughter based on the flock composition dynamics and population balance model for sheep and goat, respectively.

## Discussion

The present study estimated the extent of home slaughter and/or unregulated livestock slaughter in Iran. To our knowledge, no systematic datasets on the extent of home slaughter are available from other countries. Although anecdotal evidence suggests that home slaughter is common in many low- and middle-income settings, quantitative estimates are lacking. This underscores the novelty of our study and highlights the need for similar modeling and field-based approaches in other regions to better understand the scale of this neglected issue. Based on the models findings, 42.3–54.1% of sheep and goats slaughtered in the country, are slaughtered without the benefit of meat inspection. Unfortunately, home slaughter is a neglected issue in veterinary and agricultural sciences. Our estimates underscore that a substantial proportion of small ruminants are slaughtered outside regulated abattoirs. This finding raises critical public health concerns, particularly with respect to zoonotic infections, which represent one of the most direct consequences of unsupervised slaughter. Home slaughtering practices have been identified as significant risk factors for zoonotic disease transmission [8,29]. Due to close contact with animal carcasses and lack of veterinary supervision, the potential transmission of zoonotic infections is quite high during the slaughter of these animals. Previous studies across the globe emphasized that slaughtering out of official abattoirs is an important risk factor that facilitate the transmission of infectious diseases to human communities [10,15]. However, the literature is poor on the nature and extent of this phenomenon in different countries. This presents a knowledge gap in understanding the epidemiology and control of zoonotic infections that needs to be filled for different diseases related to home slaughter of livestock including CCHF, cystic echinococcosis, and brucellosis [8,10,11,13].

From a public health perspective, the absence of veterinary inspection during home slaughter exposes both slaughterers and consumers to several specific hazards. Brucellosis remains endemic in Iran and is transmitted through handling infected carcasses, posing one of the most significant zoonotic threats [10]. CE is a major zoonosis directly linked to unsupervised offal handling, which is happened during home slaughter. CE is perpetuated when dogs gain access to infected offal discarded after slaughter. Veterinary inspection at abattoirs normally prevents this by condemning hydatid cyst–infected organs, but during home slaughter such safeguards are absent. Infected viscera are often discarded in the environment or intentionally fed to dogs, sustaining parasite transmission. Dogs exposed to infected offal can shed *Echinococcus granulosus* eggs in the environment, contaminating pastures and water sources, which in turn infects sheep, goats, and humans [30]. Thus, CE may be considered one of the most critical and neglected consequences of home slaughter in endemic regions such as Iran.

In addition, bacterial foodborne pathogens such as *Salmonella* species may contaminate meat when slaughter is performed under unhygienic household conditions, though quantitative data from Iran are limited. Less frequently, anthrax has been reported in association with consumption of contaminated meat in Iran [12], and CCHF may be transmitted via exposure to infected animal blood during slaughter in endemic areas [13]. This highlights the diversity of risks associated with bypassing regulated abattoirs.

While these risks highlight the urgency of the issue, it is equally important to understand the underlying drivers of home slaughter. These determinants, ranging from inadequate infrastructure to cultural and economic motivations, explain why the practice persists despite its health implications. Several reasons are behind the remarkable rate of slaughtering out of official abattoirs in Iran. Inadequate infrastructures for standard industrial abattoirs, especially in the rural/ nomadic population and small cities have significant effect on high rate of home slaughter. Some centralized abattoirs in Iran, particularly in remote areas, do not fully meet international standards in terms of infrastructure, hygiene, and animal handling facilities. However, we emphasize that even in such facilities, the presence of veterinarians and meat inspectors provides a crucial safeguard for public health that is completely absent in home/ unregulated slaughter. Veterinary inspection enables detection of visible pathological lesions, condemnation of infected carcasses or viscera, and reporting of notifiable zoonoses. Thus, while upgrading abattoir facilities remains a priority, slaughter in such facilities is still preferable to home slaughter from a food safety and public health perspective [8]. Home slaughter poses significant food safety hazards, as evidenced by numerous food-borne disease outbreaks reported in Iran. A total of 2,250 foodborne disease outbreaks were reported in Iran between 2006 and 2011. Analysis of the data revealed a rising trend, with the outbreak rate increasing from 0.07 to 1.38 per 100,000 population over the six-year period [31]. A recent review highlights an increasing trend in foodborne disease outbreaks in Iran, identifying *Escherichia coli* and *Salmonella* spp. as the most frequently reported causative agents [32]. Therefore, home slaughter contributes directly to the burden of zoonotic and foodborne diseases in Iran, highlighting its significance as a One Health challenge.

Another major determinant of home slaughter is the social events in the country. In different religious/ cultural festivals, Iranian people sacrifice the animals at home as an integral part of their culture. Sacrifice as one of the fundamental parts of religious rites in human history has been practicing in most religions. There is a tradition of animal sacrifice in the Abrahamic traditions including, Judaism, and Islam as well as in Hinduism, East Asian traditions (Han-Chinese, Ancient times, Modern days), Traditional African and Afro-American religions and Austronesian [33–35]. In Iran as in other Islamic countries, Muslim communities extensively practice livestock sacrifice in Eid al-Adha as one of the most important religious festivals, commemorating the sacrifice of Abraham. It was estimated that more than 1.2 million livestock were slaughtered in Saudi Arabia during Eid al-Adha and Hajj festival [36]. In Iran according to a report in 2012 more than 23,000 small ruminants were sold in the capital city of Tehran during Eid al-Adha (Eid Ghorban, feast of sacrifice) of which 10,000 were slaughtered under veterinary supervision. This means that more than half of the livestock sold in this period were slaughtered outside official abattoirs [37]. Moreover, home slaughter is a common practice in certain social events including wedding ceremonies, funeral services as well as sacrifice and vows.

Beyond the above-mentioned cultural traditions, structural factors such as nomadism and traditional livestock husbandry also sustain high rates of unregulated slaughter in Iran. Nomadism plays a major role in livestock husbandry in Iran [27]. Nomads constitute about 1.5% of Iran population with an important contribution of livestock husbandry in the country. More than 30% of total live small ruminants (more than 37% of goat and 27% of total live sheep population) in the country belong to the nomadic people. Due to the transhumance nature of nomadic life and constant movements in remote areas, accessibility to standard abattoirs is impossible and nomads practice open slaughter. Therefore, the exact data on slaughtered animals is not available and it is difficult to estimate the rate of home slaughter in Iranian nomads. In addition to nomadism, traditional animal husbandry is common in many rural and suburban areas of the country and this increases the chance of home slaughter in these areas [38]. Nomadic mobility inherently reduces access to abattoirs, which makes home slaughter almost inevitable, and therefore increases zoonotic risks indirectly. This plays an essential role for food supply to local people and lack of veterinary supervision presents a potential risk of zoonotic infections to the communities [39–41].

**Fig 3:**
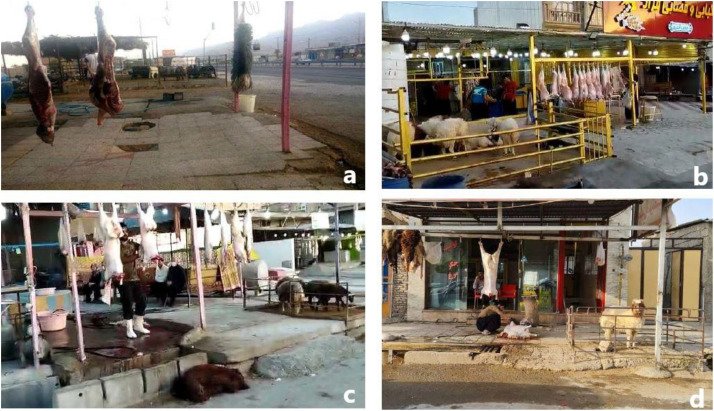
Images showing street slaughter in Iran. a) A local butchery in Bid Zard near Shiraz, Southern Iran. b, c, d) Several street butcheries in Ilam, Western Iran. These figures were created by the authors and is published under CC BY 4.0.

In addition to nomadism as a lifestyle issue, people attitude and economic incentives at the household level further reinforce the preference for home slaughter [19,42–44]. Many families believe that buying live sheep and goats is financially cost-benefit. Home slaughter offers some economic advantages for households. In Iran several families practice home slaughter and share one or two carcasses for their household meat consumption. This way the offal, as well as other edible and non-edible portions of the animals can be used with remarkably lower cost. Also, people believe this offers them a choice for direct assessment of the animal body condition. Moreover, there is a common belief that the meat freshness and quality is higher for live animals slaughtered at home or in street butcheries than those slaughtered in supervised municipal abattoirs. Fig 3 illustrates animals slaughtered in street butcheries without veterinary supervision in different provinces of Iran. Taken together, the above-mentioned factors highlight the multifactorial nature of home slaughter. Nevertheless, the accuracy of our estimates is subject to certain methodological and observational limitations, which warrant careful consideration.

Some limitations of the study could lead to some degrees of overestimations and/or underestimations. First, the quality of the underlying datasets must be considered. National-level statistics obtained from FAOSTAT and the Statistical Center of Iran (SCI) are the most reliable available sources, but like many large-scale agricultural datasets, they may be affected by underreporting, regional heterogeneity, and time lags in reporting. Unregulated slaughtering is, by nature, underrepresented in official statistics. Also, the assumptions inherent to our models introduce potential biases. The bioeconomic flock model relied on parameters that were adapted from international studies and validated against limited Iranian sources. These generalized parameters may not reflect the full diversity of Iranian production systems, especially in nomadic and peri-urban contexts.

The year 2017 was chosen for analysis because it represents the most recent year for which both FAOSTAT and SCI datasets were simultaneously available and internally consistent and the relative economic stability compared to subsequent years. After 2017, discrepancies appear between FAOSTAT and SCI reporting, likely due to differences in data collection methodologies, political and economic disruptions, and incomplete updates in national agricultural statistics. Using 2017 thus ensured that both livestock production figures and per capita meat consumption data could be reliably matched and cross-validated across the two databases. In addition, 2017 precedes the period of major economic upheavals and fluctuations in livestock trade that could further distort official reporting. Starting in late 2018, Iran experienced pronounced fluctuations in currency exchange rates, which directly impacted livestock prices, incentives for cross-border trafficking, and the reliability of reported production and consumption statistics. For these reasons, we considered 2017 to provide the most robust and internally consistent dataset for national-level modeling.

Animal transportation as a global phenomenon, is common across international and regional levels. Due to the lack of well-organized surveillance and monitoring systems for animal transportation in many countries, the rate of illegal transport of livestock remains unclear. However, because of the significant difference of meat prices between Iran and the neighboring countries, livestock trafficking is believed to be remarkable in the country. This presents a potential overestimation of home slaughter in our study. On the other hand, although comprehensive nationwide field data on home slaughter in Iran are lacking, several observational reports support our estimates. Local observations all over the country indicate a large difference between the number of animals slaughtered at local abattoirs and the number of carcasses presented in the local butcheries and meat shops. For example, in the western province of Ilam about 60 sheep and goats are usually slaughtered each day in the municipal abattoir of the city, however more than 200 animal carcasses can be recorded in 45 butcheries of the city (A.M. Bahrami, Ilam University, personal communication, Fig 3). This indicates a major underestimation of the extent of home slaughter in this region. These local data triangulate well with our model-based estimates, reinforcing the validity of our findings.

Considering the model outputs integrated with the observational reports suggests that home slaughter is indeed a widespread and neglected issue. This leads us to consider the broader implications for surveillance, control, and One Health policy. Several measures need to be implemented to resolve these challenges. Development of standard industrial abattoirs, sustainable veterinary supervision, public education, science communication, changing people attitudes towards home slaughter are among the measures necessary to reduce the impact of home slaughter in the country. Also, mobile abattoir units or temporary slaughter stations during peak religious festivals could provide flexible solutions for nomadic and remote populations.

In Iran, strengthening the capacity of the IVO to extend inspection services especially during high-demand periods, expanding networks of veterinary inspectors and meat hygiene officers, can improve compliance and consumer trust in officially supervised meat. Public awareness campaigns are necessary to highlight the risks of home slaughter, including zoonotic infections. Providing certified ‘sacrifice packages’ during cultural and/or religious festivals, where animals are slaughtered under veterinary supervision but distributed in the community, has been successful in other Muslim-majority countries. Innovative approaches have been implemented by some countries to reduce home slaughter while respecting cultural and religious traditions. Jordan and Egypt have piloted mobile and temporary slaughter stations during religious festivals, bringing basic abattoir facilities and veterinary services closer to communities to reduce informal slaughter in urban streets and rural courtyards [45,46].

In Turkey, both government authorities and humanitarian organizations have established large-scale, hygienically supervised Qurban (sacrifice) programs. For example, during Eid al-Adha 2020, a charity organization successfully managed the slaughter of over 30,000 animals across 27 countries, distributing meat to approximately 1.5 million beneficiaries, despite pandemic-related transport constraints [47]. Similarly, the Turkish Red Crescent operates an annual certified Qurban program in which donors entrust their sacrifices to be performed under veterinary and religious supervision, with the meat processed and delivered to needy families both domestically and abroad [48].

In Saudi Arabia, the Saudi Project for Utilization of Hajj Meat in collaboration with the Islamic Development Bank, centralizes sacrificial slaughter during Hajj in fully regulated abattoirs equipped with veterinary inspection, cold storage, and packaging facilities. This system allows millions of animals to be slaughtered hygienically and efficiently each year while maintaining compliance with religious requirements [49]. These initiatives demonstrate that culturally sensitive interventions, combining religious guidance, public communication, and accessible supervised facilities, can significantly decrease unsafe slaughter practices. These examples show that integrating veterinary supervision, infrastructure support, and public engagement can align cultural traditions with modern One Health principles, protecting both community health and animal welfare.

## Conclusions

Findings of the present study suggest home slaughter as a prevailing practice all over the country, claiming at least 40% of total small ruminants slaughtered in the country. Unfortunately, this is a neglected problem in veterinary and agricultural sciences. Further studies in different countries are required to clarify the nature and extent of this public health challenge across the world. Home slaughter is a multifactorial phenomenon and the measures for overcoming this challenge include a series of socioeconomic, cultural and public health programs, to make a significant change in slaughtering practice in the low- and middle-income societies.

Future research should incorporate more up-to-date and longitudinal datasets to enable the comparison across years and regions and the assessment of temporal trends in slaughter practices and their public health implications. Integrating field surveys, abattoir records, and remote-sensing or spatial data on livestock distribution could further enhance the accuracy of estimates. Such efforts would provide a stronger evidence base for monitoring progress and designing targeted interventions to reduce unregulated slaughter and its associated zoonotic and food safety risks. The multi-disciplinary “One Health” approach adopted by all stakeholders including decision-makers in various veterinary, public health and environmental sectors is desperately needed for establishing an effective monitoring and control system.

## Supporting information

S1 File SHM bioeconomic model. (XLSX)
